# Characterization of a Human Cell Line Stably Over-Expressing the Candidate Oncogene, Dual Specificity Phosphatase 12

**DOI:** 10.1371/journal.pone.0018677

**Published:** 2011-04-20

**Authors:** Erica L. Cain, Sterling E. Braun, Alexander Beeser

**Affiliations:** Division of Biology, Kansas State University, Manhattan, Kansas, United States of America; Texas A&M University, United States of America

## Abstract

**Background:**

Analysis of chromosomal rearrangements within primary tumors has been influential in the identification of novel oncogenes. Identification of the “driver” gene(s) within cancer-derived amplicons is, however, hampered by the fact that most amplicons contain many gene products. Amplification of 1q21–1q23 is strongly associated with liposarcomas and microarray-based comparative genomic hybridization narrowed down the likely candidate oncogenes to two: the activating transcription factor 6 (*atf6*) and the dual specificity phosphatase 12 (*dusp12*). While *atf6* is an established transcriptional regulator of the unfolded protein response, the potential role of *dusp12* in cancer remains uncharacterized.

**Methodology/Principal Findings:**

To evaluate the oncogenic potential of *dusp12*, we established stable cell lines that ectopically over-express *dusp12* in isolation and determined whether this cell line acquired properties frequently associated with transformed cells. Here, we demonstrate that cells over-expressing *dusp12* display increased cell motility and resistance to apoptosis. Additionally, over-expression of *dusp12* promoted increased expression of the *c-met* proto-oncogene and the collagen and laminin receptor intergrin alpha 1 (*itga1*) which is implicated in metastasis.

**Significance:**

Collectively, these results suggest that *dusp12* is oncologically relevant and exposes a potential association between *dusp12* and established oncogenes that could be therapeutically targeted.

## Introduction

Evaluation of the chromosomal region 1q21–1q23, frequently amplified in primary liposarcomas, by fluorescence *in situ* hybridization and comparative genomic hybridization reduced the list of candidate oncogenes contained by this amplicon to two genes: the activating transcription factor 6 (*atf6*) and the dual specificity phosphatase 12 (*dusp12*) [1]. ATF6 is a transcription factor involved in the unfolded protein response (UPR) which responds to endoplasmic reticulum (ER) stress [2]. While the UPR is indicated to be involved in tumorigenesis [2], the role, if any, of DUSP12 in tumorigenesis is not known. Interestingly, in four out of five liposarcomas examined, *dusp12* was expressed significantly higher than *atf6*, suggesting that *dusp12* may be the more relevant target of the 1q21–1q23 chromosomal amplification [1]. In addition, *dusp12* is over-expressed in retinoblastomas, intracranial ependymomas, and the most common childhood malignant tumor, neuroblastoma [3]–[5]. As over-expression of *dusp12* is observed in multiple cancer types, it suggests that *dusp12* may play an important role in cancer biology.

The dual specificity phosphatases (DUSPs) are members of the protein tyrosine phosphatase (PTP) family that dephosphorylate serine, threonine, and tyrosine residues [6] and are important regulators of multiple signaling pathways that modulate cell processes such as proliferation, apoptosis, and migration [7]. Misregulation of DUSPs, and hence the pathways they regulate, play a major role in the development of many diseases, including cancer and diabetes [8], [9]. Members of the DUSP family can be subdivided into subgroups based on the presence of specific domains and sequence similarity. One poorly characterized subgroup, the atypical DUSPs, do not fit into any better characterized subgroups and often do not regulate known targets of DUSPs such as mitogen activated protein kinases (MAPKs) [7].

DUSP12 is an atypical DUSP whose function in human cells is poorly understood [7]. DUSP12 was identified as a potential pro-survival phosphatase in an siRNA screen [10]. The identification of DUSP12 as a pro-survival phosphatase has been supported by experiments where transient over-expression of DUSP12 in HeLa cells protects from apoptosis in response to a variety of apoptotic stimuli [11]. Although DUSP12's function is poorly characterized in humans, DUSP12 is evolutionarily conserved, and DUSP12 homologs exist in yeast (GeneID: 854844), flies (GeneID: 32963), fish (GeneID: 573998), nematodes (GeneID: 177903), and mice (GeneID: 80915). Of these organisms, the function of DUSP12 has been best characterized in the budding yeast, *Sacchramyces cerevisiae*, where the gene is designated *yvh1* [12]. Human DUSP12 and Yvh1pshare 44% amino acid identity within their catalytic domain, this conservation extends to the essential C-terminal cysteine rich domain (59% identity) of unknown function that is only found in DUSP12 homologs. In *S. cerevisiae*, Yvh1p regulates cell growth and morphogenesis [13]. Surprisingly, these abilities do not map to the phosphatase domain, but to the C-terminal rich domain, as catalytically inactive variants or variants that lack the entire N-terminal phosphatase domain suppress the phenotypes of *yvh1Δ* strains, suggesting a phosphatase independent role for Yvh1p function [13], [14]. Importantly, ectopic expression of wild-type or catalytically inactive variants of the human *dusp12* gene in yeast also suppress the phenotypes of *yvh1Δ* strains, suggesting that the function(s) of DUSP12 and Yvh1p are evolutionarily conserved [14]. Recent work in yeast has also demonstrated that Yvh1p participates in 60S ribosome maturation in a phosphatase-independent manner [15], [16]. Although, it is clear that the human DUSP12 can functionally complement multiple phenotypes associated with *yvh1* deletion in a phosphatase-independent manner [14], whether DUSP12 functions similarly in human cells is currently unknown.

In this study, we have established for the first time, a stable cell line that selectively over-expresses *dusp12* in isolation and find that this cell line demonstrates increased cell motility, increased resistance to apoptotic stimuli, and has an increase in the transcript levels of two genes previously implicated in carcinogenesis, the proto-oncogene *c-met* and the collagen and laminin receptor *itga1*.

## Results

### Establishment of HEK293 cells stably over-expressing *gfp-dusp12* in isolation

**Figure 1:**
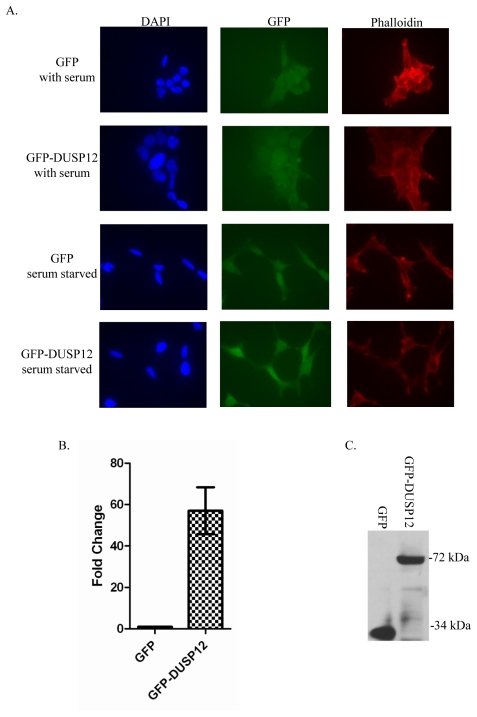
Establishment of HEK293 cells stably over-expressing either *gfp* or *gfp-dusp12* in isolation. **A**. Confocal images of HEK293 cells stably expressing GFP or GFP-DUSP12. Cells were seeded on a fibronectin coated chamber slide. After attachment, cells were washed with PBS and complete or serum free media was added to the cells. After 18 hours, cells were fixed and stained with rhodamine-phalloidin and DAPI and viewed using confocal microscopy (magnification ×100). **B**. Quantitative Real Time PCR was used to compare the expression level of *dusp12* in GFP-DUSP12 and GFP stable lines. *dusp12* specific primers were used and normalized to the average of the genes *b2m*, *actb*, and *gapd* genes. The fold change was calculated using the ΔΔCt method. The mean of three independent experiments is graphed with error bars representing SEM. **C**. Immunoblot detecting GFP or GFP-DUSP12 in HEK293 stable lines. A single clone of GFP or GFP-DUSP12 cells was lysed in RIPA buffer. Equivalent amounts of lysates were fractionated by SDS-PAGE and GFP and GFP-DUSP12 were detected with a GFP specific antibody. The blot was also probed with a total ERK 1/2 specific antibody as a loading control. Numbers to the right of the blot indicate molecular weight.

To evaluate the consequences of specific *dusp12* over-expression, we established human embryonic kidney cells (HEK293), an immortalized, but non-tumorgenic cell line [17] that stably over-expresses GFP or GFP-DUSP12 (Figure 1). Microscopic examination of the GFP-DUSP12 cell line revealed that they are morphologically similar in overall appearance to the GFP control cell line with the exception of more cortical actin present in the GFP-DUSP12 cell line (Figure 1A). In addition, there is no significant difference between the two cell lines under starvation conditions (Figure 1A). The GFP-DUSP12 cell line has close to a 60 fold increase in *dusp12* expression compared to the control GFP cell line that only expresses endogenous *dusp12* as measured by qRT-PCR (Figure 1B). Expression of the full-length GFP-DUSP12 fusion protein was confirmed by immunoblotting (Figure 1C). All the data shown were generated with the use of one individual clone designated F78; however we observed similar results in other individual clones as well as in transient expression assays, suggesting that the phenotypes observed are not due to disruption of an unknown gene caused by the insertions of *gfp-dusp12* into the genome (data not shown).

### Over-expression of *dusp12* does not promote proliferation or anchorage independent growth

**Figure 2:**
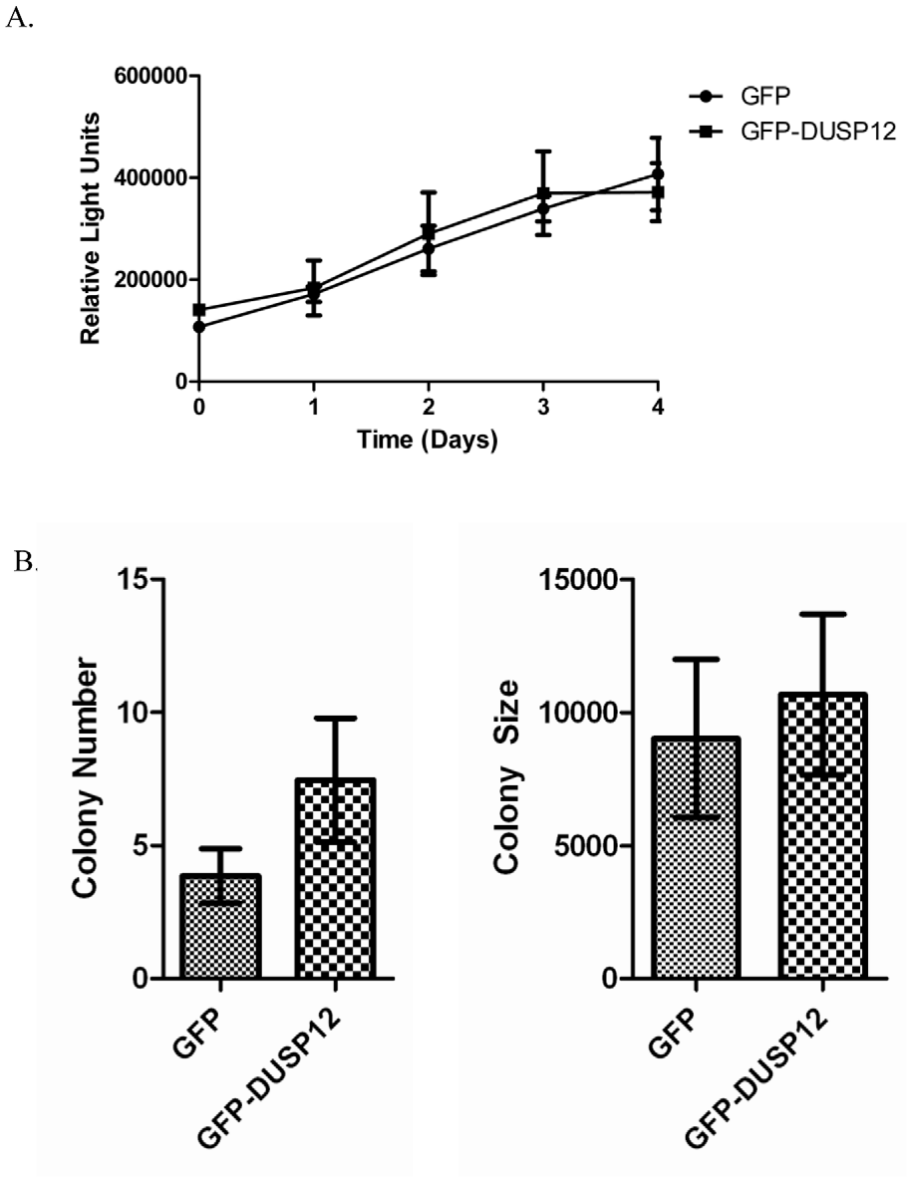
Selective over-expression of *dusp12* does not affect proliferation or anchorage independent growth. **A**. Proliferation assay was performed by measuring viable cells over time with the Promega Cell Titer-Glo assay. Time zero was the measurement of GFP or GFP-DUSP12 cells immediately after seeding the wells. The means of three independent experiments are graphed with the error bars representing SEM. **B**. GFP or GFP-DUSP12 cells were suspended in soft agar for three weeks. Colony number and size were measured using ImageJ in five fields of vision. The mean of three independent experiments are shown and the error bars represent SEM.

As *dusp12* has been described as a potential driver for the 1q21–1q23 amplicon [1] and many oncogenes promote proliferation [18], we addressed whether over-expression of *dusp12* affected proliferation. Equivalent numbers of GFP and GFP-DUSP12 cells were seeded at day 0 and proliferation was assessed as a function of time by measurement of cellular ATP levels. We found no significant difference between cells over-expressing *gfp-dusp12* compared to the cell line over-expressing *gfp* alone (Figure 2A). As anchorage independent growth is another common property of some transformed cell lines, we asked whether *dusp12* over-expression allowed for growth in soft agar. Although we noted a slight increase in the number of colonies in the GFP-DUSP12 cell line after 21 days, there was no significant difference between the GFP-DUSP12 and GFP control cell line in either colony number or size (Figure 2B).

### Over-expression of *dusp12* promotes cell motility

**Figure 3:**
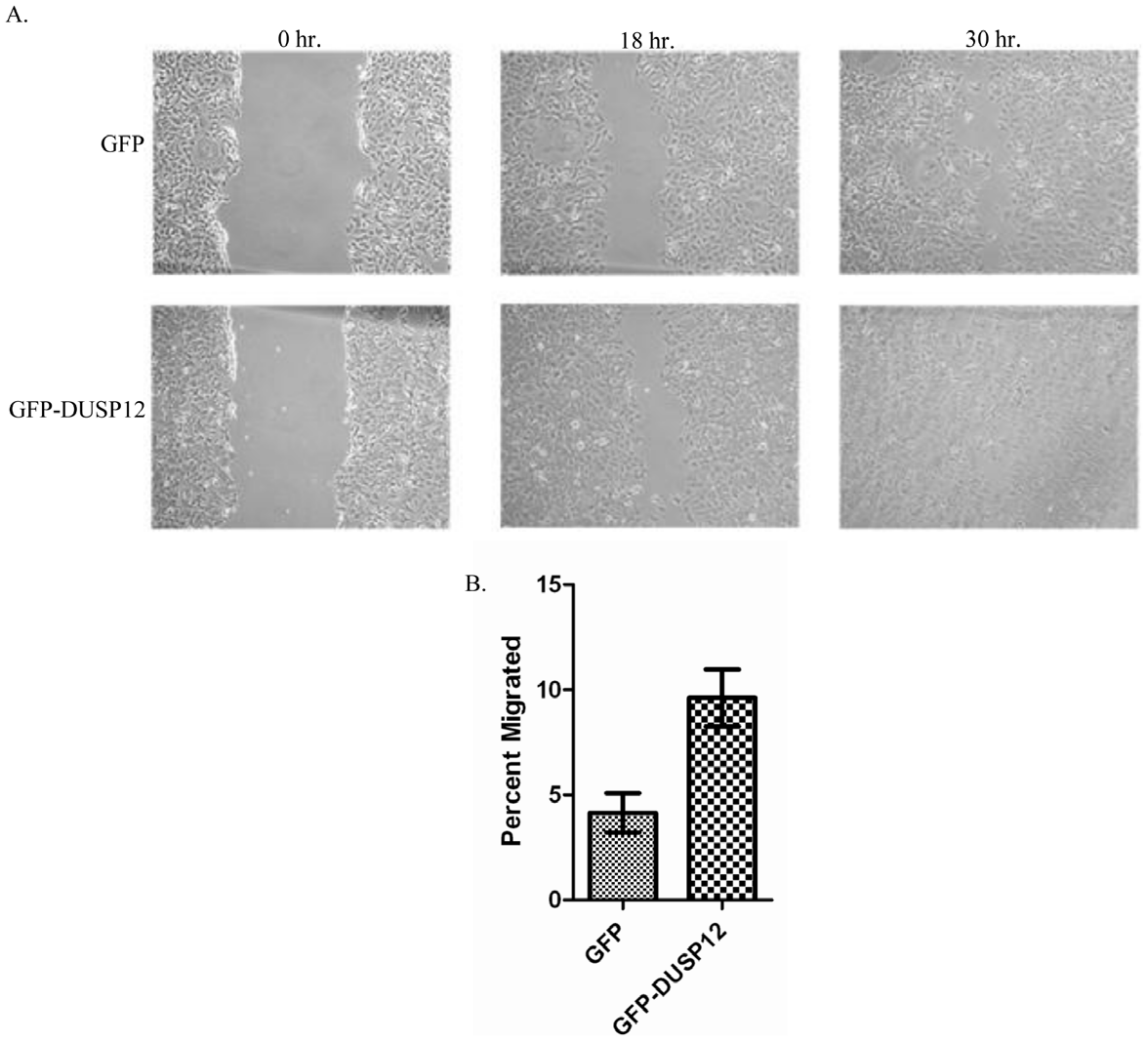
Over-expression of *dusp12* promotes cell motility. **A**. A scratch wound assay was performed on confluent GFP and GFP-DUSP12 cells and the wound closure was monitored over time. Above is a representative experiment of three independent experiments. **B**. A transmigration assay using 0.8 µm HTS Fluoroblok transmigration chambers was performed with fetal bovine serum as the chemoattractant. Cells were pre-labeled with calcein AM and added to the wells in serum free media. After 22 hours the total number of live cells was measured and the percent of total cells that migrated to the lower chamber are graphed. The means of three independent experiments are graphed with the error bars representing SEM. Significance of a P value<0.05.

Since *dusp12* over-expression is strongly associated with invasive liposarcomas [1], we tested the hypothesis that DUSP12 over-expression affects cell motility using a scratch wound assay. We observed that the GFP-DUSP12 cells were able to close the wound faster than the control cell line (Figure 3A). In order to better quantify the difference in cell motility, we conducted a transmigration assay using fetal bovine serum as the chemoattractant. The GFP-DUSP12 cell line had a statistically significant 1.2 fold increase in cell motility compared to the GFP control cell line (Figure 3B). Collectively, these results suggest that the selective over-expression of *dusp12* leads to increased cell motility.

### Over-expression of *dusp12* protects cells from apoptosis

**Figure 4:**
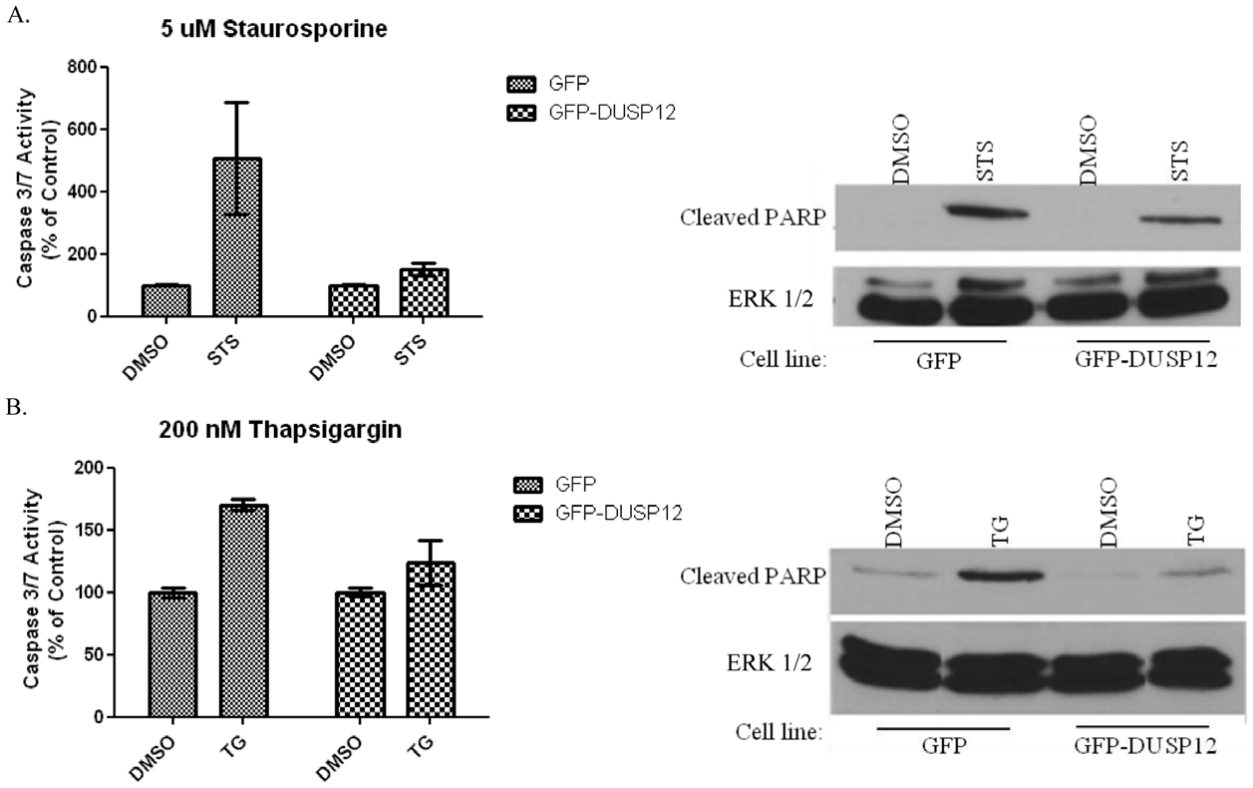
Over-expression of *dusp12* protects cells from apoptosis. **A. Left:** GFP or GFP-DUSP12 cells were treated with DMSO or 5 µM staurosporine (STS) overnight and the Promega Caspase 3/7 Glo assay was used to measure apoptosis. Means of three independent experiments are graphed with error bars representing SEM. Significance of a P value<0.05. **Right:** Immunoblot detecting cleaved PARP in lysates of GFP and GFP-DUSP12 cells treated overnight with 5 µM STS. Lysates were collected with RIPA buffer and equalized by total protein using a BCA assay. A representative blot from three independent experiments is shown. **B. Left:** GFP or GFP-DUSP12 cells were treated with DMSO or 200 nM thapsigargin (TG) for 48 hours and the Promega Caspase 3/7 Glo assay was used to measure Caspase 3/7 activation as an indication of apoptosis. Means of three independent experiments are graphed with error bars representing SEM. Significance of a P value<0.05. **Right:** Immunoblot detecting cleaved PARP in lysates of GFP and GFP-DUSP 12 cells treated with 200 nM TG for 48 hours. Lysates were collected with RIPA buffer and equalized by total protein using a BCA assay. Results depicted are representative of at least three independent experiments.

Another common property of oncogene expression is the resistance to programmed cell death. In order to determine whether ectopic over-expression of *dusp12* affected apoptosis, we treated the GFP and GFP-DUSP12 cell lines with staurosporine (STS) a broad specificity kinase inhibitor that has been widely used to induce apoptosis in a variety of different cell types. Apoptosis was quantified by means of a luminescent Caspase 3/7 assay and by immunoblotting to detect cleaved PARP, a validated apoptosis marker [19]. Treatment of the control cell line with STS led to a significant increase in both Caspase 3/7 activity (Figure 4A, leftmost panel) and PARP cleavage (Figure 4A, right panel). The GFP-DUSP12 cells demonstrated both a reduced level of Caspase 3/7 activity and PARP cleavage after STS treatment. To ensure that these results were not specific to STS-induced apoptosis, we repeated these experiments using thapsigargin (TG), which induces the unfolded protein response (UPR) by perturbation of Ca^2+^ levels [20]. Similar to STS, over-expression of *gfp-dusp12* led to a decrease in both Caspase3/7 activity and PARP cleavage induced by TG (Figure 4B). Collectively these results indicate that cells ectopically over-expressing *dusp12* have an increased resistance to apoptosis induced by different stimuli.

### Over-expression of *dusp12* up-regulates the proto-oncogene *c-met* and the metastasis factor *itga1*

**Figure 5:**
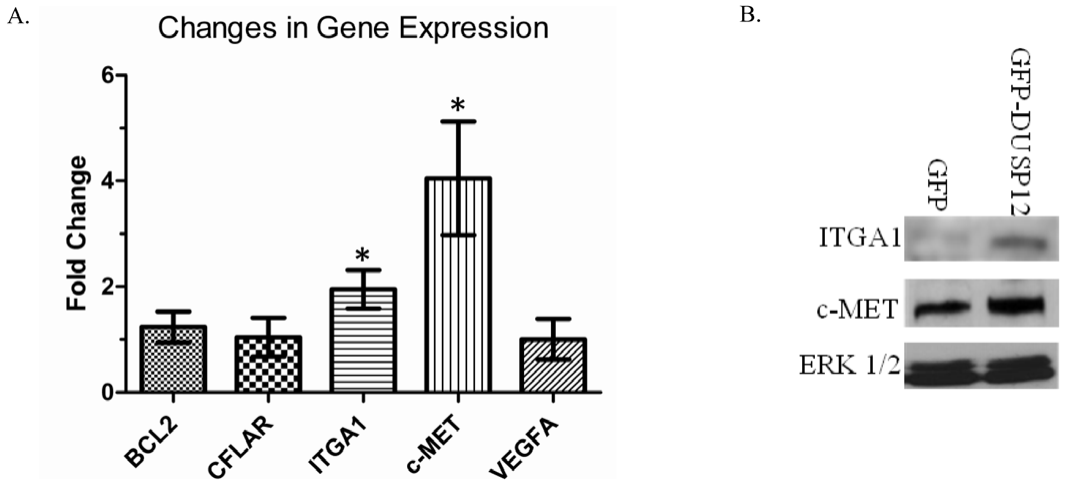
Over-expression of *dusp12* up-regulates the proto-oncogene c-MET and the metastasis factor ITGA1. **A**. The expression levels of five proto-oncogenes in GFP-DUSP12 cells were compared to the expression levels in GFP cells and normalized to the average of the housekeeping genes *b2m*, *actb*, and *gapd*. The fold change was calculated using the ΔΔCt method. The mean of three independent experiments is graphed with error bars representing SEM. * indicates P value<0.05. **B**. Immunoblot of GFP and GFP-DUSP12 cells probed with c-MET, ITGA1, and ERK 1/2 specific antibodies. Lysates were collected in RIPA buffer and equalized by total protein as measured by a BCA assay. The blot shown is a representative blot of three independent experiments.

To further characterize the consequences of ectopic *dusp12* expression, we used a Cancer Gene PCR array to compare the transcript levels of 84 genes previously implicated in transformation or tumorigenesis on RNA samples extracted from the GFP and GFP-DUSP12 cell lines. Candidate genes were identified as those demonstrating greater than a two-fold expression difference in the GFP-DUSP12 cell line compared to the GFP control cell line. This approach initially identified five candidate genes that were up-regulated by DUSP12: *bcl-2*, *cflar*, *itga1*, *vegfa*, and *c-met*. To confirm the initial PCR array results, qRT-primers for these genes were obtained and additional qRT-PCR analysis was performed using independent RNA samples extracted from the GFP and GFP-DUSP12 cell lines. This experiment confirmed that intergrin alpha 1 (*itga1*) and the hepatocyte growth factor receptor tyrosine kinase (*c-met*) transcripts were significantly up-regulated in cells over-expressing *dusp12* (Figure 5A). The increased expression was also reflected at the protein level since western blots using c-MET and ITGA1 specific antibodies demonstrated increased amounts of these proteins in GFP-DUSP12 cells compared to the GFP control cell line (Figure 5B), suggesting that the increased transcript levels found in GFP-DUSP12 cells can have biological consequences.

## Discussion

The GFP-DUSP12 cell line and the GFP control cell line were used to investigate whether over-expression of *dusp12* promotes oncogenic properties in a cell culture model. To our knowledge, this is the first time in which a stable cell line over-expressing *dusp12* in isolation has been described. This study demonstrates that over-expression of *dusp12*, may promote cancer development and progression by increasing migration and cell survival. Results obtained from the GFP-DUSP12 cell line can be replicated in transient assays where a Flag tagged DUSP12 is expressed in HEK293 cells (Figure S1), showing that GFP is not altering DUSP12 function and that the phenotypes observed are not due to clonal variation.

The up-regulation of *c-met* and *itga1* in cells over-expressing *dusp12* further indicates that *dusp12* may function as a novel oncogene, since ITGA1 is known to promote proliferation, invasion, angiogenesis, and metastasis [21]–[25], while c-MET can affect proliferation, survival, and migration [26]–[28]. The ability of DUSP12 to up-regulate c-MET and ITGA1 may explain the increased cell motility observed in the GFP-DUSP12 cell line since both of these proteins are known to increase cell migration [25], [28]. As DUSP12 is known to be over-expressed in invasive sarcomas [29], [30], and we have demonstrated that selective up-regulation of DUSP12 leads to increased *c-met* expression, it would be interesting to examine whether primary sarcomas containing the 1q21–1q23 amplicon also demonstrate increased expression of the *c-met* proto- oncogene.

In addition to regulating the expression of two genes previously implicated in various aspects of tumorgenesis, we found that *dusp12* over-expression increased resistance to apoptosis. Initially, *dusp12* was identified as a pro-survival gene in an siRNA screen using HeLa cells [10]. This was supported by other experiments where transient over-expression of *dusp12* protected HeLa cells from apoptosis induced by a variety of stimuli [11]. Our results with the immortal, but non-tumorigenic, HEK293 cell line suggest that DUSP12 over-expression can promote apoptosis resistance is the third instance of *dusp12* being described as a pro-survival gene, and suggests that DUSP12 can protect from apoptosis in a variety of cellular contexts. The ability of DUSP12 to promote apoptosis resistance may be due to the up-regulation of c-MET which has been described to promote cell survival [27]. However, we were unable to detect c-MET activation by immunoblotting with an antibody specific to c-MET phosphorylated at Tyr 1234/1235 (data not shown), suggesting that DUSP12 may protect from apoptosis in a manner independent of c-MET activation. Investigations into mechanisms by which DUSP12 can protect from apoptosis are ongoing.

*c-met* is over-expressed in many different neoplastic diseases [31]. Studies have found that over-expression of *c-met* imbues cells with properties of cellular transformation, however, properties such as anchorage independent growth require the c-MET ligand, hepatocyte growth factor (HGF), supplied either in an autocrine or paracrine manner [26], [27], [29], [30], [32]–[34]. Our inability to observe c-MET activation, growth in soft agar, or increases in proliferation in the GFP-DUSP12 cells may be due in part to the low expression of HGF by HEK293 cells [33]. Although growth in soft agar is often used as a metric for cellular transformation, not all oncogenes are capable of promoting anchorage-independent growth. Notably, over-expression of the *bcl-2* oncogene in normal rat epithelial cells (WBF443) and JB6 mouse epidermal cells, does not affect growth rate nor does it allow for anchorage independent growth [35], [36]. We speculate that in the correct cellular context and microenvironment DUSP12's ability to up-regulate c-MET could result in cellular transformation. For reasons that are not well understood despite repeated efforts we were able to recover cells that only stably over-express GFP, but not the GFP-DUSP12 fusion, from a human fibrosarcoma (HT1080), cervical adenocarcinoma (HELA), breast adenocarcinoma (MCF-7) or a mouse fibroblast (NIH3T3) cell line (data not shown).

At this time, investigations are ongoing that are examining how DUSP12 can cause the phenotypes described here. Of interest is whether the phosphatase or the unique cysteine rich domain of DUSP12 is required for the up-regulation of genes and increases in survival and cell motility. Previous work in HeLa cells suggests that the pro-survival function of DUSP12 is dependent on phosphatase activity [11]. However, Yvh1p affects yeast growth [13], [14] and promotes 60S ribosome maturation [15], [16] in a phosphatase independent manner. Both protein translation and ribosome biogenesis are coordinated with cell proliferation, and interfering with these processes can retard cell growth and animal development [37]. A role for ribosome biogenesis in cancer progression comes from the recent observation that c-MYC localizes to nucleoli where it functions as a regulator of ribosome biogenesis [38]. Whether DUSP12 contributes to ribosome biogenesis in mammalian cells and if so, to what extent, awaits further investigation.

In summary, we describe for the first time the establishment of a cell line that over-expresses the *dusp12* gene in isolation and demonstrate that these cell lines are endowed with several cancer relevant properties: increased motility, resistance to apoptosis, and up-regulation of two genes (*c-met* and *itga1*) which are implicated in transformation and/or metastasis. As *dusp12* is present within the 1q21–1q23 amplicon present in primary liposarcomas and other tumor types, this study suggests a possible role for DUSP12 in cancer progression.

## Materials and Methods

### Plasmids and plasmid construction

The human *dusp12* cDNA in plasmid pOTB7 was obtained from OpenBiosystems. This plasmid was used as a template for polymerase chain reaction using Pfusion polymerase (NEB) with the following oligonucleotides incorporating 5′ BamHI and 3′ EcoRI restrictions sites 5′-GCCCGGATCCATGTTGGAGGCTCCG-3′ and 5′-GCGAATTGTCATATTTTTCCTGTT-3′. The resulting PCR fragment was digested with BamHI and EcoRI and ligated into the baculoviral transfer vector pFastBacHTB (Invitrogen) similarly digested. The clone was then confirmed by sequencing the entire length of the insert. The DUSP12 fragment was digested with BamHI/EcoRI and ligated in to pEGFP-C1 (Clontech) digested with BglII/EcoRI to create plasmid pEGFP-DUSP12.

### Immunoblotting

Cells were lysed in radioimmunoprecipitation assay buffer (RIPA) containing 1 mM phenylmethylsulfonyl fluoride, 10 µg/ml aprotinin, and 1 mM sodium orthovanadate (Fisher). Cleared cell lysates were obtained by centrifugation (21000× g for 10 min at 4°C) and standardized by total protein as measured by a BCA assay (Pierce). Equivalent amounts of cleared lysates were fractionated by SDS/PAGE and transferred to PVDF membranes (Millipore). The membranes were blocked in 5% fat free milk in Tris-buffered saline containing 0.1% Tween 20 (TBST) for at least 1 hour at room temperature. All primary antibodies were diluted in blocking buffer at 1∶1000 and incubated rolling at 4°C overnight. Primary antibodies used in this study are GFP (Cell Signaling #2555), Cleaved PARP (Cell Signaling #9541), p44/p42 MAPK (ERK 1/2) (Cell Signaling # 4695), ITGA1 (Abcam #ab78479), and MET (Cell Signaling #3127). HRP- linked secondary antibodies were from Cell Signaling (#7074 and #7076) and used at a dilution of 1∶30000 in blocking buffer and incubated at room temperature for one hour. For signal detection the Immobilon Chemiluminescent HRP substrate was used as recommended by the manufacturer (Millipore),and signals were obtained by exposing blots to X-ray film (MIDSCI).

### Cell culture

HEK293 cells were obtained from ATCC (CRL-1573). All growth media, serum and supplements were purchased from Hyclone. HEK293 cells were grown in 1× Eagle's Minimal Essential Medium (EMEM) supplemented with 10% fetal bovine serum and penicillin/streptomycin. The GFP and GFP-DUSP12 HEK293 stable lines were cultured in the complete HEK293 media with the addition of 800 µg/ml G418 (Fisher). All experiments using HEK293 or derivative cell lines were conducted in the absence of G418 except the soft agar assays. Cells were grown at 37 °C, 5% CO_2_ in a humidified chamber.

### Establishment of GFP and GFP-DUSP12 stable cell lines

pEGFP-C1 or pEGFP-DUSP12 were transfected into HEK293 cells using the TransIT-293 transfection reagent (Mirus) in a 6 well plate following the manufacturer's recommendations. 24 hours later, cells were washed in phosphate buffered saline (PBS), trypsinized, and placed in one 100 mm tissue culture dish, and allowed to attach. Complete media was removed and replaced with complete media containing 800 µg/ml G418. Every two days the G418 containing media was replenished. After approximately 16–20 days of selection, individual clones were isolated using cloning discs, and individually expanded followed by screening for the recombinant GFP-DUSP12 or GFP expression by immunoblotting with a GFP specific antibody.

### Immunofluorescence

GFP or GFP-DUSP12 cells were seeded at 40–50% confluence in Millipore 8 chambered slides coated with 200 µl of 50 µg/ml fibronectin (Sigma). After over-night attachment, cells were washed in PBS, and complete media or serum free media was added to the cells and incubated. After 18 hours the cells were washed in PBS, fixed in 3.7% formaldehyde in PBS for 15 minutes at room temperature, washed with PBS, and permeabilized using 0.2% Triton X in PBS for 10 minutes at room temperature. Cells were labeled with rhodamine-phalloidin as described by the manufacturer (Invitrogen). Cells were washed three times in PBS and 100 ng/ml DAPI (Roche) was added for five minutes prior to addition of ProLong antifade reagent (Invitrogen). Images were taken at 100× using a Zeiss confocal microscope.

### Quantitative real time PCR

Total RNA was isolated from GFP and GFP-DUSP12 stable cell lines cultured in complete media, using the RNAeasy kit (Qiagen) including the optional on the column DNAse treatment. The quality of total RNA for each sample was verified by the Agilient 2100 Bioanalyzer. cDNA was produced using the RT^2^ First Strand kit as described by manufacturer (SABiosciences). Quantitative real time PCR was performed using the RT^2^ SYBR Green Master Mix following the manufacturer's protocol (SABiosciences) using an iCylcer iQ Real Time PCR Detection System (BioRad). Fold change was calculated using the ΔΔCt method. Expression levels of *dusp12* were monitored using d*usp12* specific primers described in [39]. qRT-PCR primers for housekeeping genes (*actb*, *b2m*, and *gapd*) were obtained from RealTimePrimers.com. To screen for cancer relevant genes that are up or down regulated by DUSP12 over-expression, we used a cancer relevant PCR array from SABiosciences (PAHS-033) as described by the manufacturer. The array contains 84 genes that are known to be involved in tumorigenesis. Genes identified from the arrays with at least a two-fold change were then verified by qRT-PCR done in triplicate with primers for the *c-met*, *itga1*, *vegfa*, *cflar*, and *bcl2* genes from SABiosciences.

### Proliferation assay

GFP or GFP-DUSP12 cells were counted using trypan blue exclusion and a hemacytometer. 250 cells in 75 µl were seeded per well of a 96 well plate. Viable cell number was monitored over time using the Cell Titer Glo kit as described by the manufacturer (Promega).

### Soft agar assay

1.2×10^4^ GFP or GFP-DUSP12 stable cells were suspended in 1 ml of 0.3% Difco Noble Agar (BD) in Dulbecco's Modified Eagle Medium (DMEM) supplemented with 10% fetal bovine serum, penicillin/streptomycin, and 800 µg/ml G418, and then added to a six-well plate with a foundation layer of 0.5% agar in triplicate. 24 hours later, the cells received 1 ml of complete medium containing G418 before incubation for 21 days. Complete media was replenished at least once a week to prevent drying. Colonies were stained with .005% crystal violet (Fisher) dissolved in phosphate buffer saline solution for one hour at 37 C. Five fields of vision at 4× magnification were taken for each well and analyzed for colony number and size by ImageJ (NIH).

### Caspase activity

Viable cells were counted using trypan blue exclusion and a hemacytometer. Approximately 2×10^4^ cells were added per well to a 96 white walled clear bottom plate. The next day, either staruospoine (STS) or thapsigargin (TG) (Acros Organics) were added to the cells at a final concentration of 5 µM STS or 200 nM TG, and incubated for an additional 16 hours or 48 hours respectively. For both treatments addition of DMSO (Fisher) serves as a vehicle control. Following incubation with STS or TG, the Promega Caspase 3/7 Glo assay was performed as described by the manufacturer. Luciferase activity was measured using a Perkin-Elmer Victor 3V. For immunoblots examining cleaved PARP, cells were seeded in a 100 mm plate and treated with a final concentration of 5 µM STS, 200 nM TG, or DMSO and lysates were collected in RIPA buffer at the indicated times.

### Cell motility

For the scratch wound assay, GFP or GFP-DUSP12 cells were placed on a coverslip in a six well plate. Once cells reached confluence, a wound was created by using a micropipette tip. Pictures of the same area of the scratch were taken at 10× magnification at the indicated times. Each assay was performed in triplicate. For the transmigration assay, GFP or GFP-DUSP12 cells were pre-labeled with Calcein AM as described by the manufacturer (BD). Following labeling, cells were suspended in serum free media and placed in a HTS Fluorblok transwell in a 24 well format (BD). Complete medium was used as a chemoattractactant. Cells were also placed in a well of the 24 well plate without a transwell to be used to measure total fluorescent signal of the cells. Fluorescence was read at 0 and 22 hours using a Perkin Elmer Victor 3V. The signal at 0 hours of the transwell was subtracted from the 22 hour read. Percent migration was calculated by dividing the background subtracted 22 hour transwell signal by the 22 hour signal from the control wells that contained no transwell. Each assay was done in triplicate.

### Statistics

P values were obtained using either a standard two tailed t-test or a two- way ANOVA with Bonferroni Post-Test using GraphPad Prism.

Supporting materials and methods are described in File S1.

## Supporting Information

Figure S1 **Transient expression of Flag tagged DUSP12 in HEK293 cells promotes cell motility and up-regulation of c-MET.** **A. Left:** A transmigration assay using 0.8 µm HTS Fluoroblok transmigration chambers was performed with fetal bovine serum as the chemoattractant. At 24 hours post transfection, cells were pre-labeled with calcein AM and added to the wells in serum free media. After 22 hours the total number of live cells was measured and the percent of total cells that migrated to the lower chamber are graphed. The means of three independent experiments are graphed with the error bars representing SEM. **Right:** Immunoblot of lysates from HEK293 cells transiently expressing Flag tagged DUSP12 or the empty vector. Blot was probed with an anti-Flag antibody (Sigma #F3165). Immunoblot shown is representative of three independent experiments. **B. Left:** Immunoblot of lysates from HEK293 cells transiently expressing a Flag tagged DUSP12 or the empty vector. Blot was probed with antibodies specific to c-MET, ERK 1/2 (loading control), and Flag. Immunoblot shown is representative of three independent experiments. **Right:** Densitometry was performed using ImageJ. The fold change compared to the empty vector control is graphed after normalization with the loading control (ERK 1/2). Graphed are the results of three independent experiments with the error bars representing SEM.
(TIF)
