# Supplementary figures and images for: Characterization of a Human Cell Line Stably Over-Expressing the Candidate Oncogene, Dual Specificity Phosphatase 12
Source: PLoS One. 2011 Apr 20;6(4):e18677. doi: 10.1371/journal.pone.0018677 (PMC3080379; doi:10.1371/journal.pone.0018677)

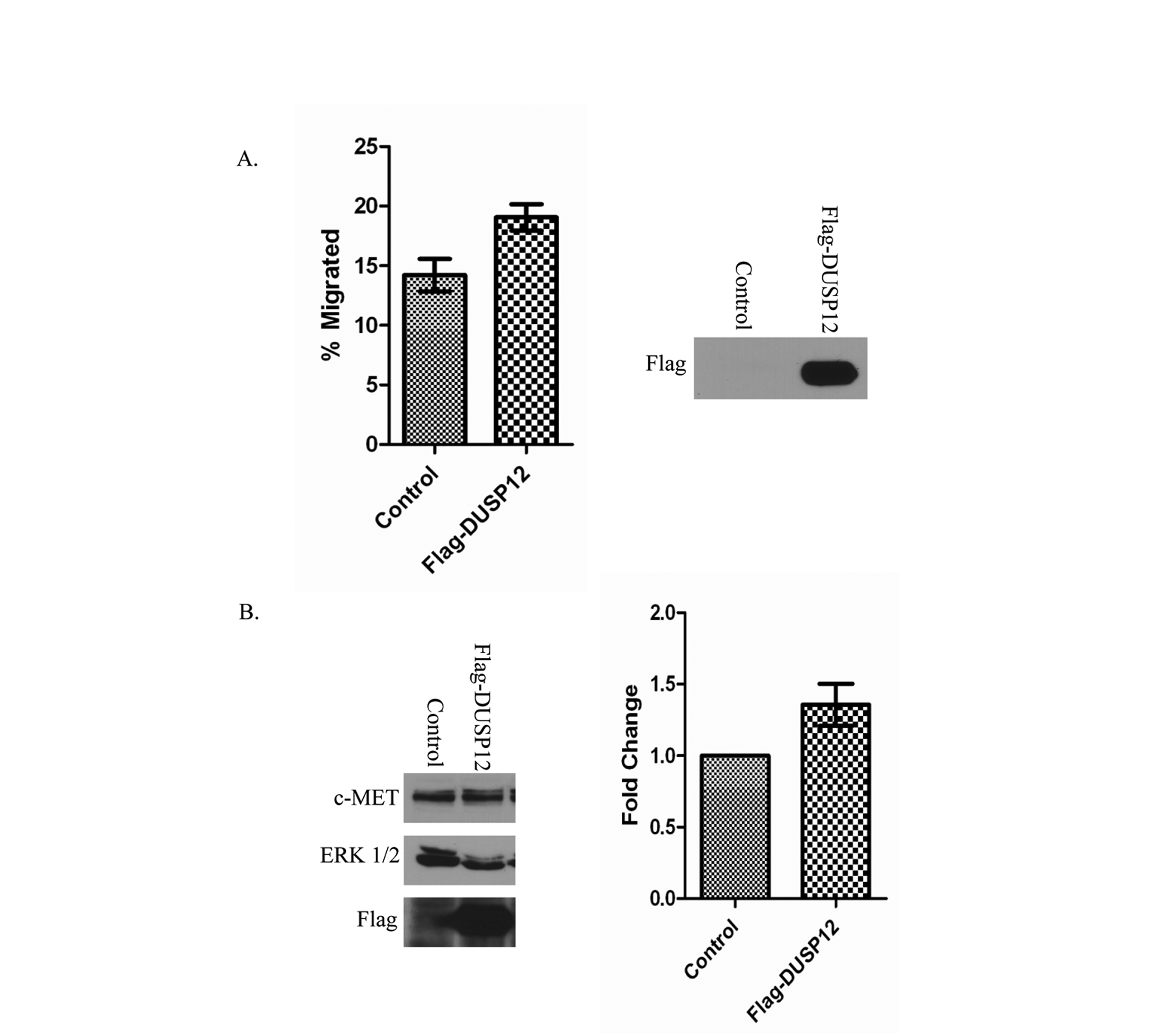

Supplement: Figure S1 — Transient expression of Flag tagged DUSP12 in HEK293 cells promotes cell motility and up-regulation of c-MET. A. Left: A transmigration assay using 0.8 µm HTS Fluoroblok transmigration chambers was performed with fetal bovine serum as the chemoattractant. At 24 hours post transfection, cells were pre-labeled with calcein AM and added to the wells in serum free media. After 22 hours the total number of live cells was measured and the percent of total cells that migrated to the lower chamber are graphed. The means of three independent experiments are graphed with the error bars representing SEM. Right: Immunoblot of lysates from HEK293 cells transiently expressing Flag tagged DUSP12 or the empty vector. Blot was probed with an anti-Flag antibody (Sigma #F3165). Immunoblot shown is representative of three independent experiments. B. Left: Immunoblot of lysates from HEK293 cells transiently expressing a Flag tagged DUSP12 or the empty vector. Blot was probed with antibodies specific to c-MET, ERK 1/2 (loading control), and Flag. Immunoblot shown is representative of three independent experiments. Right: Densitometry was performed using ImageJ. The fold change compared to the empty vector control is graphed after normalization with the loading control (ERK 1/2). Graphed are the results of three independent experiments with the error bars representing SEM. (TIF) [file pone.0018677.s001.tif]
